# Exploiting *in situ* NMR spectroscopy to understand non-traditional methods for zeolite synthesis†

**DOI:** 10.1039/d4sc07931k

**Published:** 2025-02-06

**Authors:** Nicole L. Kelly, Emma A. L. Borthwick, Gaynor B. Lawrence, Paul S. Wheatley, Colan E. Hughes, Kenneth D. M. Harris, Russell E. Morris, Sharon E. Ashbrook

**Affiliations:** a School of Chemistry, EaStCHEM and Centre of Magnetic Resonance, University of St Andrews North Haugh St Andrews KY16 9ST UK rem1@st-andrews.ac.uk sema@st-andrews.ac.uk; b School of Chemistry, Cardiff University Park Place Cardiff CF10 3AT UK HarrisKDM@cardiff.ac.uk

## Abstract

Zeolite-formation mechanisms have long been the subject of intensive study, with most work concentrating on hydrothermal mechanisms. However, non-traditional zeolite syntheses that do not rely on hydrothermal crystallisation have provided a number of new routes to interesting and unexpected new materials, but their formation mechanisms remain poorly understood. Here, we show how simultaneous *in situ* liquid- and solid-state ^29^Si NMR spectroscopy can reveal the mechanism of the formation of a zeolite from a layered silicate precursor. The study provides evidence for the species that are intercalated into the layered material and establishes those that are involved in building the inter-layer, zeolitic connections as a function of time during the zeolite formation process.

## Abstract 2

Kinetic and mechanistic aspects of the intercalation step in non-traditional zeolite synthesis are studied using simultaneous liquid- and solid-state *in situ* NMR spectroscopy of isotopically enriched materials.

## Graphical abstract

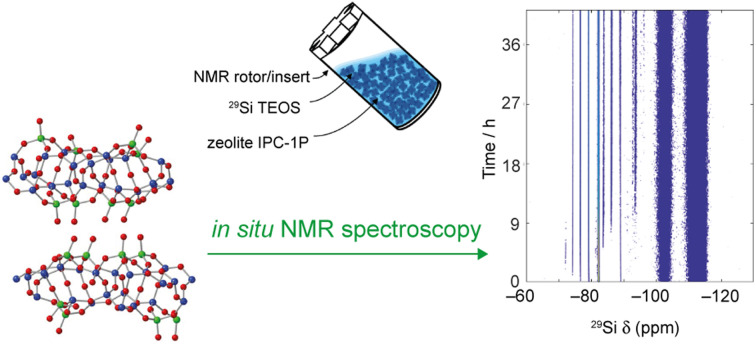

## Introduction

Silicate-based zeolites are one of the most important classes of porous materials, primarily because of their use in industry as adsorbents and catalysts.^1^ The specific application of a zeolite depends on the pore size, which controls the substrates that can interact with the internal surface of the material. The synthesis of zeolites with new topologies and different pore sizes therefore remains an important target in modern science, together with understanding the mechanisms of zeolite formation. The traditional method of zeolite preparation uses hydrothermal crystallisation.^2^ However, over the last two decades several new synthetic methods have been identified, including 1D to 3D topotactic condensation of chain silicates,^3^ inverse sigma transformation of germanosilicate^4^ and the thermal condensation of layered silicates.^5,6^ These developments have further led to the intriguing possibilities of mixed zeolite materials^7^ prepared from exfoliated layered silicates^8^ and condensation routes to form high-energy, so-called unfeasible, zeolites.^9^

Among the non-traditional routes for generating novel zeolites, the ADOR (Assembly, Disassembly, Organisation, Reassembly) process has generated significant interest.^6,10^ This involves the preparation of a parent zeolite, which has inherent weakness engineered into its structure, which results in a selective disassembly to generate stable building units. After an organisation process which can involve the relative rearrangement of these units with respect to each other, the (re-)intercalation of silica from solution or the deliberate addition of organising agents, such as structure directing agents, the building blocks can be reconnected or reassembled, leading to a new daughter zeolite.^6,10^ The ADOR process is complex, dependent on the exact conditions used and the rates at which the different steps of the process take place, leading to the possibility of producing a number of different daughter zeolites from the same parent. Both powder X-ray diffraction (XRD) and NMR spectroscopy have been used to attempt to characterise the starting materials, intermediates and final daughter products.^6,10–16^ Powder XRD is particularly useful for characterising the more crystalline initial parent and final daughter zeolites. Although long-range order is often partially or completely lost in the intermediate materials formed during the reaction, powder XRD can still provide useful information on, *e.g.*, the spacing of the zeolitic layers in the intermediates present.^14^ In contrast, the sensitivity of NMR spectroscopy to the atomic-scale environment makes it an ideal tool for studying local structure, disorder and (in particular) chemical reactivity in the solid state,^17–19^ and it has been extensively employed for the study of microporous solids more generally,^20–26^ and specifically to follow the structural changes taking place in the ADOR process.^10–16^ The majority of NMR studies of the ADOR process have focussed on ^29^Si (*I* = 1/2) NMR spectroscopy,^10,11,13,14,16^ where the ratio of Q^4^ (*i.e.*, Si connected *via* oxygen to four other Si) to Q^3^ (where one O–Si is replaced by O–H) species is often characteristic of particular intermediate phases. Furthermore, ^17^O NMR spectroscopy has been used to show the dynamic behaviour of both layered zeolites^12,16^ and fully connected zeolite frameworks^27^ when in contact with water.

In this work, we exploit NMR spectroscopy both to characterise the intermediates and products formed in the ADOR reaction and to follow this process *in situ*, gaining new information directly on the mechanism and kinetics. Specifically, we consider the reaction between a silicon-containing liquid, tetraethyl orthosilicate, Si(OEt)_4_ (or TEOS), and the layered silicate zeolite IPC-1P – an example of the organisation (or “O”) step of the ADOR process. IPC-1P is typically produced from the controlled disassembly of a Ge-UTL parent zeolite^10^ by the selective hydrolysis of the Ge that occupies the d4r between the zeolite layers, in the “D” or disassembly step of the ADOR process. This leads to a layered Ge-free material where all the d4r have been removed. Intercalation of silicate species into IPC-1P forms the partially connected IPC-2P^10^ intermediate (or COK-14 (ref. 4)) without further crystallisation or thermal calcination steps (an organisation or “O” step). A fully condensed zeolite, IPC-2, where the silicate zeolite layers are joined by s4r, can be formed from IPC-2P after a high temperature reassembly (or “R”) step.^10–14^ The use of TEOS as an intercalating agent is well established, with examples including intercalation into clays, silicates and layered double hydroxides to form new types of catalysts and adsorbents,^28,29^ into MXenes to form new battery electrodes,^30^ and into carbon materials to form new pillared solids with controllable gallery size.^31^ The common feature of these processes, whatever the material, is the proposed mechanism, in which the intercalating species is TEOS itself, and any further reaction occurs between the layers within the material (a so-called intragallery or interlamellar reaction^32^).

*In situ* NMR spectroscopy provides an ideal approach to follow the structural changes that take place directly during a reaction and has recently been applied to study the formation of porous solids.^33,34^ To understand the mechanism of the process(es) taking place in the organisation reaction described above, it is necessary to simultaneously probe both the liquid- and solid-state components present in the reaction. This can be achieved by acquisition of separate “liquid-state” and “solid-state” spectra, using either interleaved acquisition (as in CLASSIC NMR^35–37^) or phase encoding and post processing (as in SASSY NMR^38^). Conventionally in these approaches, solid-state NMR signals are selectively observed using cross polarisation (CP^39^) and the transfer of magnetisation, usually from ^1^H, *via* the dipolar coupling that is suppressed by rapid motion in solution. However, the inherently non-quantitative nature of this technique (reliant on the internuclear distances), poses a problem for the current work, where quantitative measurements of the proportion of Q^*n*^ Si species is desired. Here, we discriminate between species in the liquid and solid states using their differential relaxation (with interleaved acquisition of ^29^Si NMR spectra using shorter and longer recycle intervals), although the challenges here are discussed in detail below. Owing to the low natural abundance of ^29^Si (4%), which would limit spectral sensitivity and impact the time resolution possible when following the *in situ* reaction, we also exploit isotopic enrichment of both the starting layered silicate and the TEOS itself. Modelling the changes in the intensities of all signals as a function of reaction time provides direct insight into the species present at each point in the reaction and allows the rate constants and mechanism of the reaction to be determined.

## Methodology

### Synthesis of ^29^Si-enriched UTL

A gel of composition 0.8 : 0.4 : 0.4 : 38 (SiO_2_ : GeO_2_ : ROH : H_2_O) was prepared by dissolving amorphous germanium dioxide (0.609 g, 5.82 mmol, 99.999% Acros) in a 0.584 M solution of the SDA, (6*R*,10*S*)-6,10-dimethyl-5-azoniaspiro[4,5]decane hydroxide (10 mL, 5.84 mmol). Tetraethyl orthosilicate at natural abundance (2.094 g, 10.05 mmol, 98% Aldrich) was added dropwise along with ^29^Si-enriched tetraethyl orthosilicate (0.333 g, 1.59 mmol, >99 at% ^29^Si CortecNet), before stirring at room temperature for 2 h. The solution was transferred to a Teflon-lined autoclave (23 mL, Parr Instruments) and heated at 175 °C for 13 days under static conditions. The solid was recovered by filtration, washed with copious amounts of distilled water and dried overnight. To remove the occluded SDA, the zeolite was heated to 575 °C at a rate of 1 °C min^−1^, held at 575 °C for 6 h and cooled to room temperature at a rate of 2 °C min^−1^ under an atmosphere of air. The final product had a Si/Ge ratio of 4.4 (determined by EDX) and a ^29^Si enrichment level of ∼18%.

### Synthesis of ^29^Si-enriched IPC-1P

^29^Si-enriched IPC-1P was synthesised by hydrolysing 1 g of calcined ^29^Si-enriched (18%) Ge-UTL in 200 mL of 0.1 M HCl at 90 °C for 16 h under reflux. The product was filtered, washed with water, and dried at 80 °C overnight.

### Solid-state NMR spectroscopy

Solid-state NMR spectra were acquired using a Bruker Avance NEO instrument, equipped with a 20 T wide-bore magnet, operating at a Larmor frequency of 168.9 MHz for ^29^Si. A powdered sample of IPC-1P (∼15 mg, 18% ^29^Si) was mixed with 15 μL of ^29^Si-enriched (99%) TEOS in a PTFE HRMAS insert, which was placed inside a 4 mm ZrO_2_ rotor and rotated at a MAS rate of 5 kHz, using a conventional Bruker HXY probe. All ^29^Si chemical shifts are shown in ppm relative to Si(CH_3_)_4_, using the OSi(CH_3_)_3_ resonance of octakis(trimethylsiloxy)silsequioxane (Q_8_M_8_) (*δ* = 11.5 ppm) as a secondary reference. Spectra were acquired at 50 °C, with this temperature pre-calibrated using methanol.

For the *in situ* experiments, interleaved acquisition of ^29^Si MAS NMR spectra with recycle intervals of 1 s (averaging 128 transients) and 30 s (averaging 16 transients) was performed, to acquire the liquid- and solid-state spectra separately over 40 hours, using 90° (3.2 μs) pulses with a radiofrequency nutation rate of ∼78 kHz. Note that the first 5–10 min of the reaction is not able to be accessed owing to the time required to insert and spin the sample, and to tune the probe. See Section S1 of the ESI† for a more detailed discussion of relaxation and quantitation in these systems. Under these measurement conditions the liquid-state signals are observed in both sets of spectra. However, as described below, the signals from the liquid- and solid-state components are generally well resolved and have limited overlap. Therefore, detailed analysis was performed for signals from both phases in the spectra acquired using the longer recycle interval only. We note that a similar approach has been used previously^40^ to monitor the evolution of crystallization of an organic material by *in situ*^13^C NMR spectroscopy, with signals observed for both liquid-state species and solid-state species in the same type of spectrum; in this case, the solid phase has substantial molecular motion. In the present work, it should also be noted that subsequent experiments performed at 14.1 T gave similar results, confirming the reproducibility of the observations. However, due to the lower sensitivity of these measurements, only the results obtained at 20.0 T are discussed here. Two sets of data were considered; the first contained each spectrum in the data set, and the second contained “binned data” (where three consecutive spectra were combined to improve sensitivity, but with the loss of time resolution). In the main text, analysis of only the second set of data is shown, but similar analyses for the first set of data are shown in Fig. S5.4 and Table S5.2 of the ESI,† along with a brief discussion of the fitting employed and the uncertainties in the measurements in Section S2.†

## Results and discussion

**Fig. 1:**
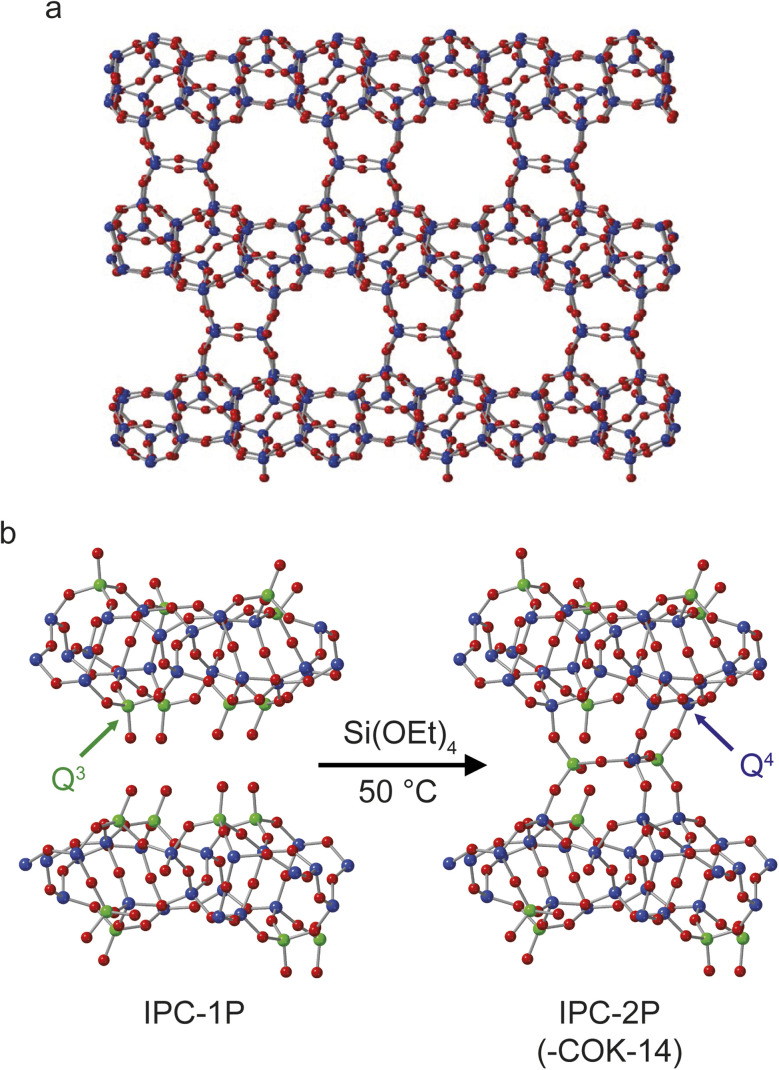
Schematics showing (a) the structure of IPC-2/COK-14 and (b) the process of forming IPC-2P through rection of TEOS with the layered silicate IPC-1P. Red atoms are oxygen, green are Q^3^ silicon and blue are Q^4^ silicon. An expanded view of one layer of the structure of IPC-1P is given in Fig. S3.1 of the ESI.†

Fig. 1 shows the structures of IPC-2 (COK-14), IPC-1P and IPC-2P. Although IPC-2 could be deemed the ultimate final product of the reaction of IPC-1P with TEOS, it is unlikely that this reaction will go to completion under the conditions used and on the timescale studied. The overall process probed in this study, therefore, is the transformation of IPC-1P to IPC-2P, shown in Fig. 1b. IPC-1P^8^ is prepared by controlled disassembly of Ge-UTL, a parent germanosilicate zeolite which comprises silicate-rich layers linked by germanosilicate double four rings.^41–43^ When these are hydrolysed under aqueous conditions, the resulting IPC-1P layered silicate is held together by hydrogen bonding between the silanol groups that line the interlayer surfaces. These groups, as shown in Fig. 1b, are arranged in octets, with four silanols on the layer above and four on the layer below. These octets are ideally aligned to reconnect if extra silicon is intercalated between the layers and condenses to form inter-layer O–Si–O linkages, as shown in Fig. 1b. The large pore nature of the parent UTL is retained in the IPC-1P layers, meaning that the octets are well separated and can essentially be treated as independent sets of reaction sites that do not interfere with each other. Fig. 1 also shows the colouring used in this work to denote Q^4^ (**Si**(OSi)_4_, blue) and Q^3^ (**Si**(OSi)_3_(OH), green) Si species. In later figures, Q^2^ (**Si**(OSi)_2_(OH)_2_) Si species are shown in yellow.

**Fig. 2:**
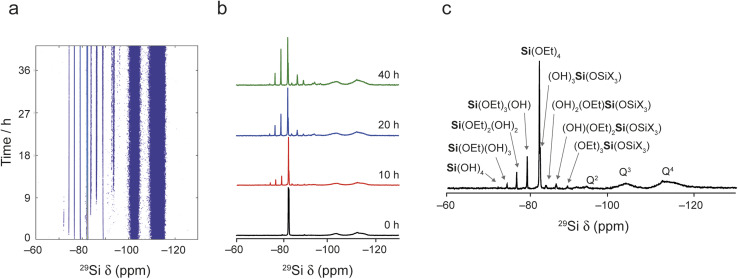
^29^Si MAS NMR spectra (20. 0 T, 5 kHz) recorded *in situ* during the reaction of IPC-1P and TEOS loaded into a HR MAS insert inside the NMR rotor. (a) Intensity contour plot of the spectra (acquired using a 30 s recycle interval) as a function of time. (b) Spectra extracted from (a) at 0, 10, 20 and 40 h showing the varying signal intensities. (c) Spectrum resulting from a sum projection of the spectra in (a) onto the horizontal axis together with the suggested assignment of each signal (see also Fig. S5.2 and Table S5.1†).

Fig. 2 shows the *in situ*^29^Si NMR spectra recorded as a function of time, with a recycle interval of 30 s. The NMR spectra show two sets of signals: sharp signals arising from mobile ^29^Si species in the liquid, and broader lineshapes resulting from ^29^Si species in the solid (for ^29^Si MAS NMR spectra of the starting materials prior to reaction see Fig. S5.1 of the ESI†). The signals from the liquid and solid are generally well separated (in the ranges from −70 to −95 ppm and −95 to −120 ppm, respectively), with only a very small extent of overlap of signals from low-level oligomeric species in solution (see below) with the Q^2^ signal from the solid. Given this, the detailed analysis below is carried out only on the experiments acquired with the 30 s recycle interval.

Fig. 2 shows that, while TEOS (at *δ* = −82 ppm) is the only Si species present in the liquid state at the start of the reaction, new liquid-state signals resulting from the hydrolysis of TEOS appear as the *in situ* reaction progresses. The signals at higher shift result, as shown in Fig. 2c, from Si species with increasing numbers of attached OH groups, with **Si**(OEt)_3_(OH) at −79 ppm, **Si**(OEt)_2_(OH)_2_ at −76 ppm, **Si**(OEt)(OH)_3_ at −74 ppm and **Si**(OH)_4_ at −72 ppm (assigned by reference to previous literature).^44,45^ Note that the spectrum shown in Fig. 2c is obtained from a sum projection of the two-dimensional dataset in (a) onto the horizontal axis to enable different signals present at different times during the reaction to be seen in a single spectrum. This does not represent the real relative intensities of the signals seen at any one point in the reaction (spectra recorded at 0, 10, 20 and 40 h of reaction are shown separately in Fig. 2b).

At longer reaction times, low levels of oligomeric species are seen at lower chemical shift, which can be assigned to species such as (OH)_3_**Si**OSiX_3_ at −82 ppm, (OH)_2_(OEt)**Si**OSiX_3_ at −84 ppm, (OH)(OEt)_2_**Si**OSiX_3_ at −86 ppm and (OEt)_3_**Si**OSiX_3_ at −89 ppm (where X is either OH or OEt).^44,45^ The latter signal is also seen in the TEOS starting reagent itself and likely results from a low level dimerisation to give (OEt)_3_SiOSi(OEt)_3_. At later stages of the reaction low levels of oligomeric Q^2^ species are also seen overlapping with the broader signal from the solid material. See Fig. S5.2 and Table S5.1 of the ESI† for the suggested assignment of these species.^45^

**Fig. 3:**
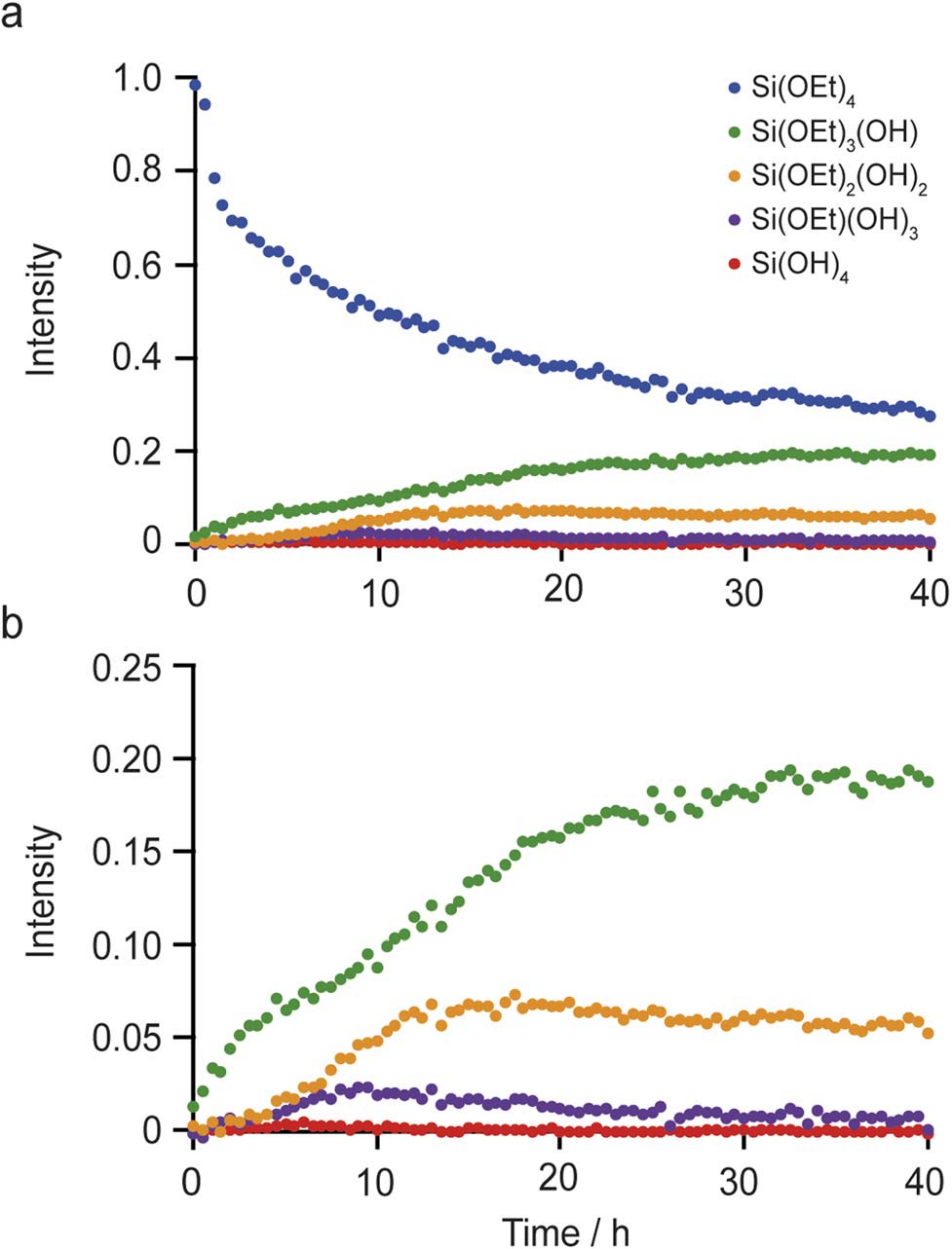
(a) Plot showing the intensities of the ^29^Si liquid-state NMR signals for TEOS and all monomeric hydrolysis products formed in the reaction with IPC-1P as a function of time. (b) Expanded view of the data in (a), highlighting the very low concentration of Si(OH)_4_. The data are normalised such that the total intensity of the monomeric species at *t* = 0 is equal to 1 (*i.e.*, the low levels of oligomeric species present are neglected).

**Fig. 4:**
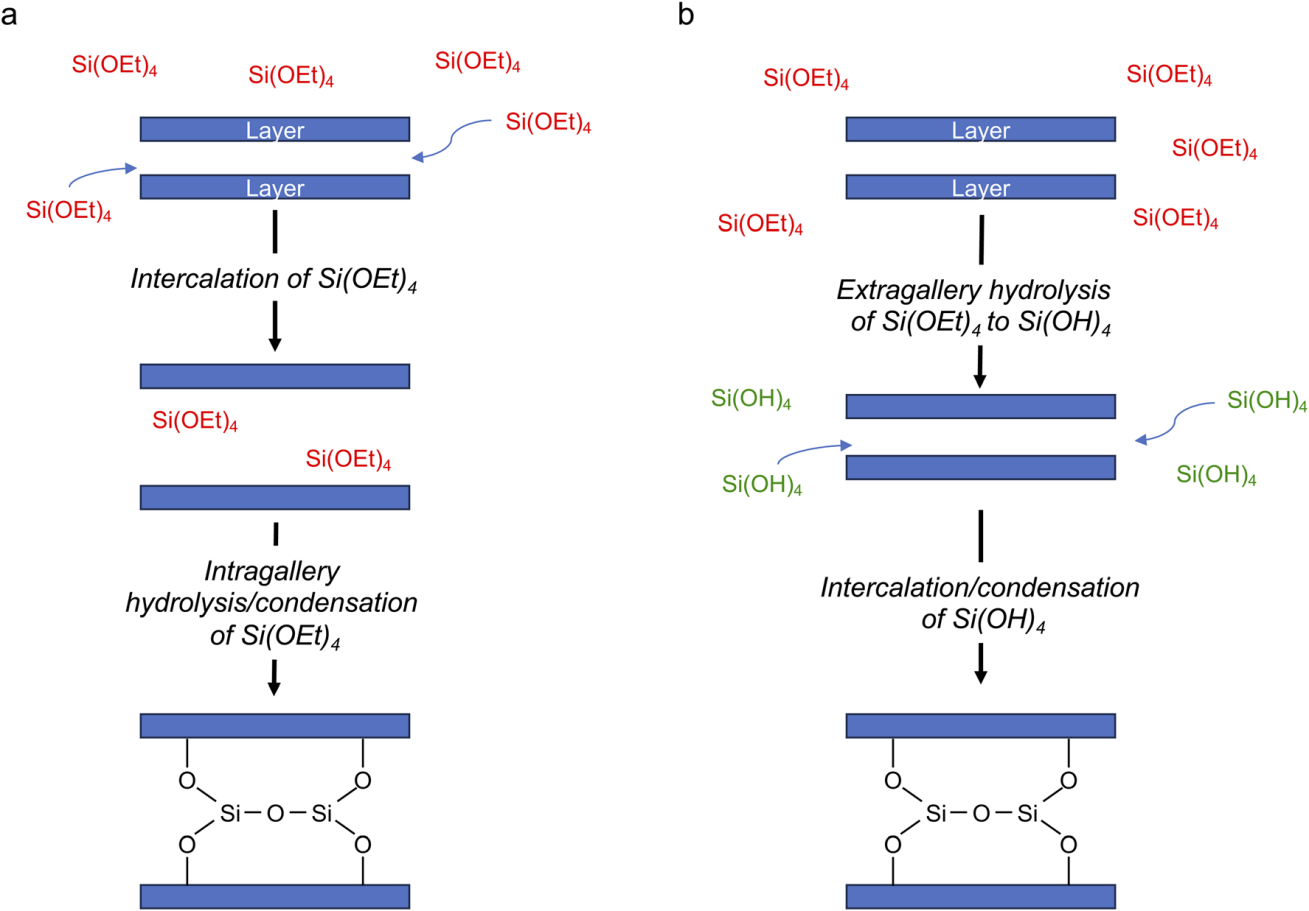
Schematic of two possible mechanisms for intercalation of silicon species in a layered host, with (a) intragallery and (b) extragallery hydrolysis.

The changes in the relative intensities of the ^29^Si signals corresponding to the monomeric hydrolysis products observed in the *in situ* reaction between TEOS and IPC-1P are plotted in Fig. 3. Although the hydrolysis of TEOS itself has been studied previously,^44,45^ this is usually in acidic solutions (and ultimately leads to gelation *via* extensive oligomerisation). It could be considered surprising that the TEOS hydrolysis occurs in the liquid phase (instead of occurring after the intercalation of TEOS into the layered zeolite where it can react with the solid acid). The hydrolysis shown in Fig. 3 proceeds in a similar way to that seen in acidic solution except that (i) relatively little formation of oligomers is seen when compared *e.g.*, to ref. 44 and (ii) the amount of Si(OH)_4_ observed is considerably less. In the work of Fyfe and Aroca in ref. 45, when the amount of TEOS had been reduced to 50% of its starting value there was ∼30% of Si(OH)_4_ present. In Fig. 3b it can be seen that the amount of Si(OH)_4_ present in the *in situ* reaction with IPC-1P is always less than ∼1%, with a maximum concentration observed at *ca.* 6–8 h. In principle, the fact that only a low amount of Si(OH)_4_ is observed could be due to the formation of condensation products in the liquid phase (as seen in Fig. 2), although the amount of these products observed here is too low to account completely for this effect. At the end of the experimental measurements (*i.e.*, after 40 h of reaction) the total liquid-state ^29^Si NMR signal (including all oligomers) is ∼10% lower than at the start of the reaction, and (although not strictly quantitative between solution and solid) this is accompanied by an increase in the intensity of the solid-state signals in the spectrum (as shown in Fig. 2 and S5.3 of the ESI†). Taken together, this evidence strongly suggests that the major species intercalated within IPC-1P is Si(OH)_4_, rather than TEOS itself. This seems feasible as Si(OH)_4_ is the smallest molecule present and could be assumed to be most easily incorporated between the layers, and it would also interact most favourably with the hydrophilic silanol groups that line the layers. However, this assignment contrasts with previously published mechanisms for intercalation reactions, in which TEOS is described as the intercalating species, followed by intragallery hydrolysis, as described above.^32^ Schematics for these two possible hydrolysis mechanisms are compared in Fig. 4. Further evidence supporting the hydrolysis of TEOS (and the subsequent production of ethanol) in solution, rather than hydrolysis after intercalation into the zeolitic layers, can be seen in the ^1^H NMR spectra shown in Section S7 of the ESI.† Note that these spectra are dominated by liquid-state signals as the solid-state ^1^H signals are much broader, particularly at the MAS rate used. The spectra show the production of ethanol in solution as the TEOS hydrolyses (Fig. S7.1†) and reveal that the total intensities of the CH_3_ and CH_2_ signals (summed for TEOS and ethanol) remain essentially constant throughout the reaction (Fig. S7.2†). This suggests that there is relatively little intercalation of TEOS itself into the zeolite (for which significantly broader signals would be expected as a result of the restricted motion once the molecule is confined and/or bound) but suggests the hydrolysis of TEOS to Si(OH)_4_ which is then intercalated. These ^1^H NMR spectra support the hydrolysis of TEOS occurring primarily in solution, followed by the intercalation of Si(OH)_4_ rather than of TEOS itself. Future *in situ* and *ex situ*^1^H NMR studies would be possible in the future (but ultimately may be limited by both the spectral resolution and the restricted MAS rate).

**Fig. 5:**
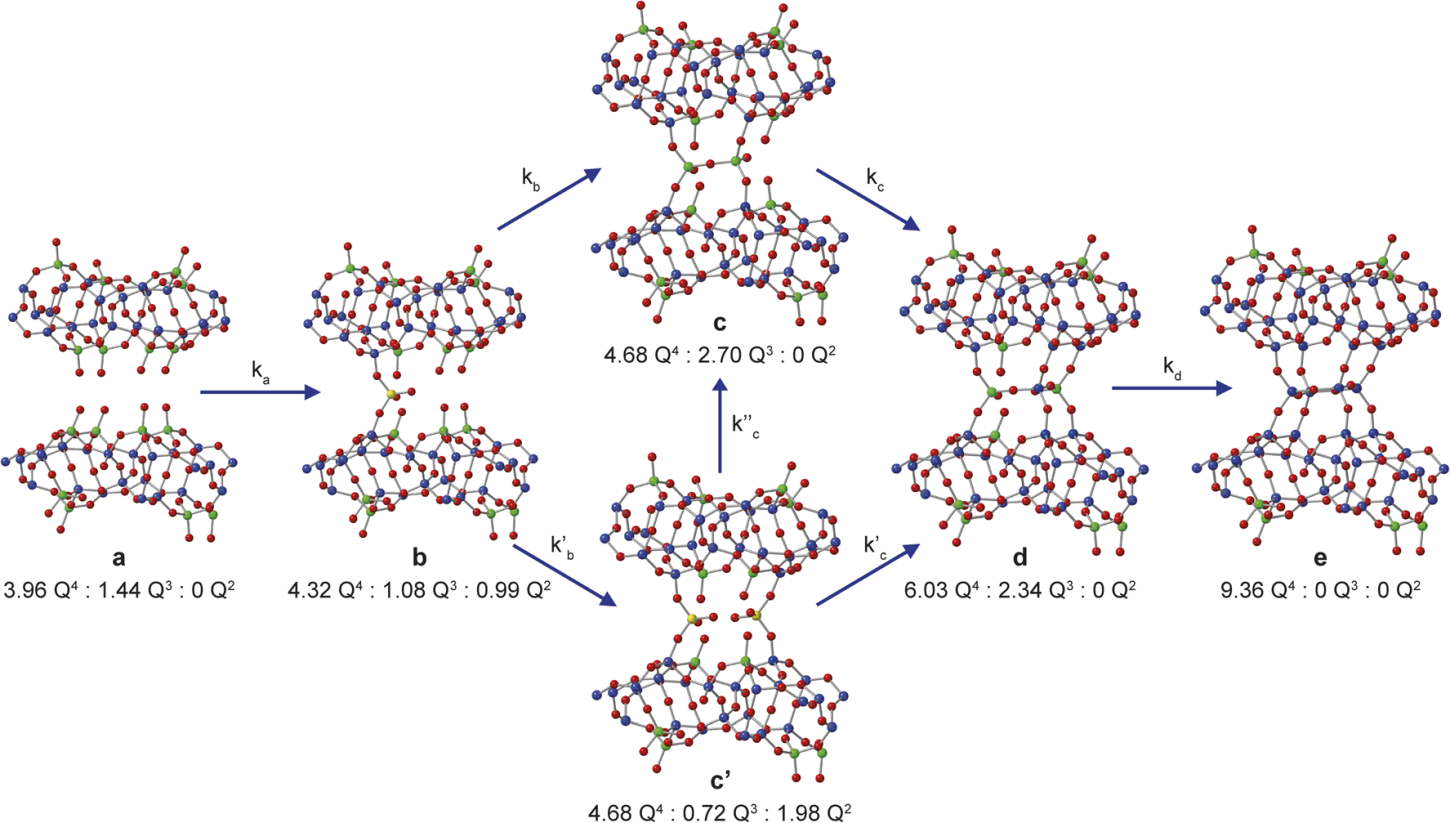
Schematic showing the proposed mechanism of intercalation of Si(OH)_4_ into the layers of IPC-1P. The ratios of NMR-active ^29^Si Q^4^, Q^3^ and Q^2^ species for each of the structural models a, b, c, c′, d and e are given. Oxygen atoms are shown in red, and Q^2^, Q^3^ and Q^4^ Si atoms in yellow, green and blue, respectively.

Assuming that Si(OH)_4_ is the only species that intercalates between the zeolitic layers, the mechanism and kinetics of this process can be considered in more detail by following the changes in intensity of the broad signals attributed to Q^2^, Q^3^ and Q^4^ species in the solid, as shown in Fig. 5. An ideal (*i.e.*, low defect) IPC-1P starting material has a well-defined ratio of Q^4^ to Q^3^ Si species, with no Q^2^ species present. As described above, the Q^3^ silanol groups in IPC-1P are arranged in well separated octets which can be treated as independent sets of reaction sites. Given the 18% isotopic enrichment of the starting material this gives ^29^Si Q^4^ : Q^3^ : Q^2^ of 3.96 : 1.44 : 0 for the structure labelled a in Fig. 5 (*i.e.*, a model IPC-1P). As Si(OH)_4_ is intercalated between the layers, it can condense to form an Si–O–Si linkage (producing a Q^1^ species) with the release of a molecule of water, and it can then react to form a second Si–O–Si linkage, generating a Q^2^ species and a second water molecule. There is no evidence for Q^1^ species with restricted mobility in the NMR spectra, which would give rise to broad signals in the range −70 to −80 ppm, suggesting that partially condensed species are not seen and rapid reaction to form a Q^2^ species occurs. As discussed above, the IPC-1P silanol octet is ideally aligned to facilitate this rapid formation of a Q^2^ species, and once Si(OH)_4_ reacts with one silanol, it is enthalpically favourable to further condense to form a Q^2^ species from the two Q^3^ silanols in the octet, and entropically favourable to release the second water molecule, leading to structure b in Fig. 5. This observation is also supported by preliminary DFT calculations (see Section S6 of the ESI†), although a complete computational study is clearly out of the scope of the current work. Geometric constraints prevent the newly formed Q^2^ species from forming any further bonds with the silanol octet. Considering the different levels of ^29^Si isotopic enrichment of the zeolite (18%) and TEOS (99%) this gives Q^4^ : Q^3^ : Q^2^ of 4.32 : 1.08 : 0.99 for the structure labelled b in Fig. 5 (see Section S5 of the ESI† for a more detailed discussion of the how the Q^4^ : Q^3^ : Q^2^ ratios are calculated for each structure in Fig. 5).

The reaction of a second Si(OH)_4_ molecule with the octet can take place at two possible positions; adjacent to the existing Q^2^ species, forming two Q^3^ Si species in the interlayer space after subsequent condensation with the proximate Q^2^ Si (structure c in Fig. 5), or at the opposite side of the octet, which results in a second Q^2^ species (structure c′ in Fig. 5). The corresponding Q^4^ : Q^3^ : Q^2^ ratios would be 4.68 : 2.70 : 0 for c and 4.68 : 0.72 : 1.98 for c′. Reaction of a third Si(OH)_4_ molecule with either c or c′ leads to product d in Fig. 5, which has Q^4^ : Q^3^ : Q^2^ of 6.03 : 2.34 : 0. Binding of the final Si(OH)_4_ forms the fully condensed structure e with Q^4^ : Q^3^ : Q^2^ of 9.36 : 0 : 0, which would correspond to IPC-2/COK-14 if repeated throughout the material. Under the conditions of the *in situ* reaction, it is unlikely that structure e would be formed (because, based on previous work^10^ the formation of e requires higher temperatures), and we would expect to form an IPC-2P(COK-14) like material as the product, which would correspond locally to structure d.

From Fig. 5 it is possible to derive expressions for the relative amounts of Q^4^, Q^3^ and Q^2^ species, giving1
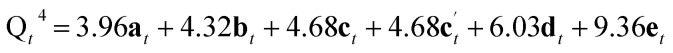
2
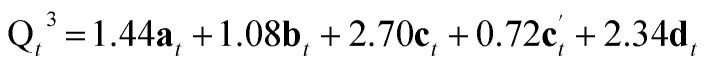
3
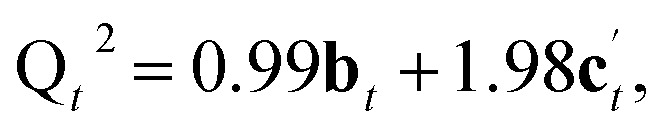
where **x**_*t*_ is the relative amount of structure **x** present at time *t*, such that4
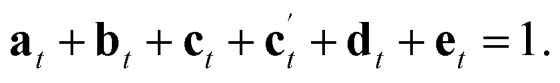
At time *t* = 0 (*i.e.*, a_*t*_ = 1) these equations reduce to Q^4^ = 3.96 and Q^3^ = 1.44, reflecting the expected ratio of species in idealised IPC-1P.

Two possible pathways for the intercalation mechanism were modelled: one going through structure c and one through structure c′. An Avrami-Erofe'ev (JMAK) type kinetic approach was employed,^46^ which has been used extensively to study transformations in the solid state, ranging from *in situ*^47^ and *ex situ*^14^ X-ray diffraction studies of zeolite formation to the kinetics of drug delivery by deintercalation from layered double hydroxides.^48,49^ The Avrami–Erofe'ev equation is

5 **x**_*t*_ = 1 − exp(−*kt*^*n*^),

where **x**_t_ is the relative amount of species **x** at time *t*, *k* is a rate constant and the exponent *n* gives information on the dimensionality and nucleation properties of the process. The value of *n* can range from 0 to 4, with *n* < 1 characteristic of diffusion-controlled reactions with decreasing numbers of reaction sites and *n* = 4 characteristic of three-dimensional interface-controlled processes with sporadic or random nucleation.^46^

**Fig. 6:**
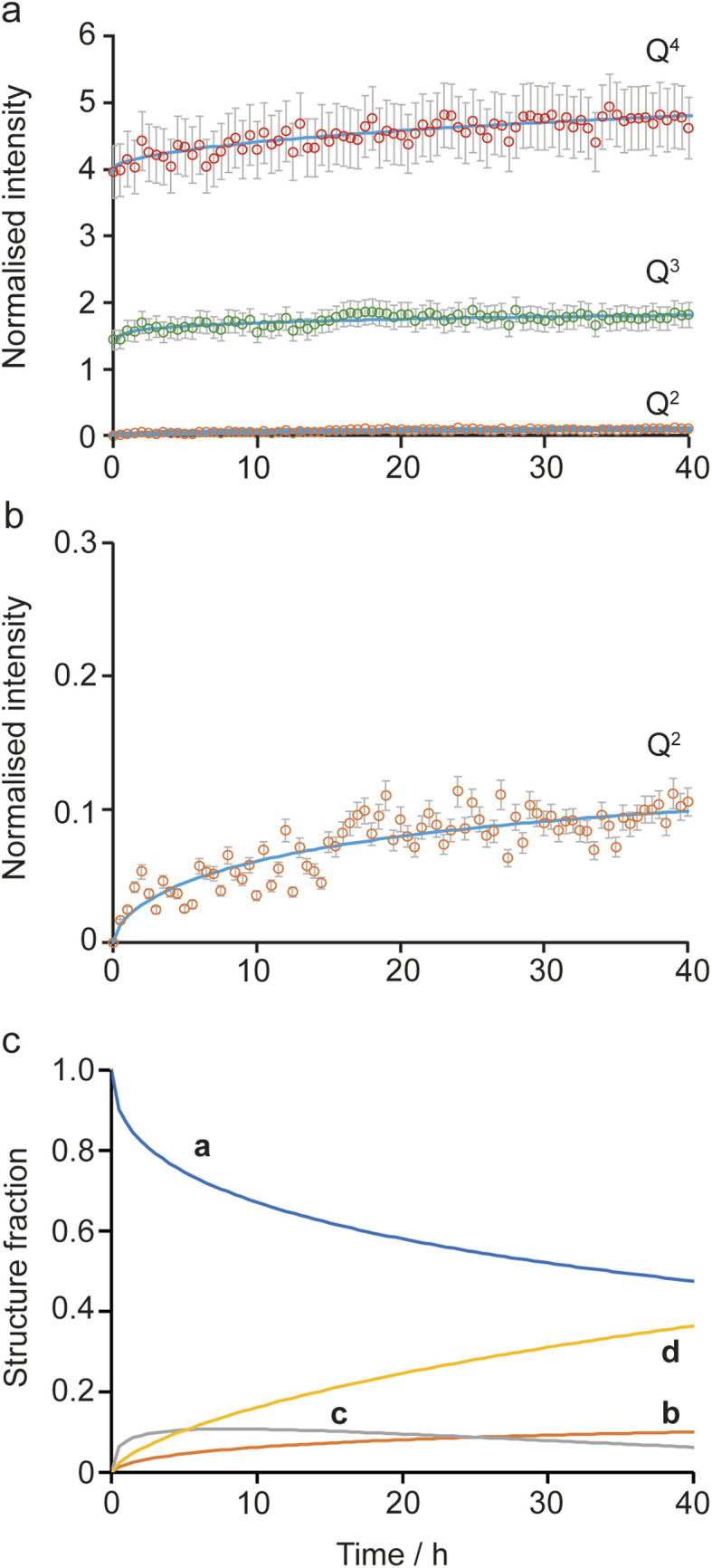
Kinetic analysis for the a to b to c to d pathway of the reaction mechanism shown in Fig. 5. (a and b) Plots showing the fitting of the intensities of the ^29^Si Q^4^, Q^3^ and Q^2^ signals calculated using the Avrami–Erofe'ev equation (blue lines) to the (normalised) experimental intensities (shown as circles with error bars) extracted from the *in situ*^29^Si NMR spectra recorded as a function of time in Fig. 2a. The estimated error bars are ±10% of the experimental value. (c) Plot showing the relative amounts of a, b, c and d as a function of time.

Initially, the successive reaction of a to b to c to d was modelled, with the three steps described by the rate constants *k*_a_, *k*_b_ and *k*_c_ and the associated exponents *n*_a_, *n*_b_ and *n*_c_. These six parameters were varied to minimize the difference between the observed (normalised) intensities of the (solid state) Q^4^, Q^3^ and Q^2^ signals in the ^29^Si NMR spectra, giving the fits shown in Fig. 6a and b, with the relative amounts of a, b, c and d present as a function of reaction time shown in Fig. 5c. The values of the rate constants and exponents for each step are given in Table 1. Although care should be taken not to overinterpret the Avrami–Erofe'ev parameters,^46^ particularly regarding the mechanistic interpretation of the exponents, *n*_i_, it is important that the values obtained from fitting should reflect, at least to some degree, the physical processes involved and should therefore make chemical sense.

**Table 1:** The best fit Avrami–Erofe'ev parameters (with estimated uncertainties) for the fits shown in Fig. 6. The units of *k*_i_ are h^−*n*_i_^

Parameter	Value
*k*_a_	0.141(3)
*n*_a_	0.452(6)
*k*_b_	0.119(3)
*n*_b_	0.419(6)
*k*_c_	0.038(4)
*n*_c_	0.67(4)

The values of the rate constants *k*_a_ and *k*_b_ in Table 1 indicate that the loss of a (to form b) is only slightly faster that the depletion of b (in this case to form c), which is consistent with the overall rate being controlled by the initial intercalation of the Si(OH)_4_ molecules into the IPC-1P layers. The initial intercalation requires that the entire layer in the IPC-1P starting material is perturbed, which is likely to have a relatively large energy barrier. However, once this has happened, the zeolite-like porosity of the material means that diffusion and condensation of the second Si(OH)_4_ is more straightforward, although some steric hindrance at any given octet might be envisaged. This suggests a layer-by-layer reaction similar to that proposed for other intercalations, including in the ADOR process.^11^ The values of *n*_a_ and *n*_b_ are close to 0.4, consistent with an intercalation reaction where the number of reaction sites decreases with time, and consistent with results from previous work on zeolites using *ex situ* diffraction techniques.^14,50^ The formation of d is significantly slower than the preceding steps, with *k*_c_ smaller than *k*_a_ and *k*_b_ by over an order of magnitude (see Table 1). The value of *n*_c_ (0.67) is a little higher than *n*_a_ and *n*_b_ but still below 1. While the diffusion of Si(OH)_4_ molecules into the material through the zeolitic porosity should be similar in this step, the fact that two sites in an octet are already occupied by intercalated silicon species in structure c impinges on the ease with which further Si(OH)_4_ molecules can find a free site at which to condense in the octet. As shown in Section S5 of the ESI,† very similar results are obtained using the unbinned dataset.

An alternative pathway for the reaction shown in Fig. 5 involves the formation of intermediate c′ rather than c, significantly reducing the number of Q^3^ sites present at this stage of the reaction. For this pathway, it was not possible to find as good a fit to the experimental data using the Avrami–Erofe'ev equation, with the prediction of unphysical results (such as negative quantities of structural models c or d). Attempts to fit intermediate mechanisms involving both c and c′ were also unsuccessful. One possible reason why the formation of c may be favoured over c′ is simple statistics – when starting from b, addition of Si(OH)_4_ at two of the three vacant sites in the octet leads to the formation of c, while addition at only one site leads to c′. It is also worth considering the dynamic nature of the interlayer space. Previous work on the mechanism of the ADOR and inverse sigma zeolite transformation processes has shown that silicon atoms in the interlayer space are more dynamic than those in the layers.^12,50^ This suggests that even if c′ is formed initially on intercalation, it could then rapidly rearrange to form c (as shown in Fig. 5), which is both enthalpically and entropically favourable as it maximises the condensation reactions and the production of molecular water. If this rearrangement were faster than the timescale of the NMR experiments, c′ would not be observable in the NMR spectra as it would not persist long enough to be measured.

The possibility of adding the fourth Si(OH)_4_ molecule (*i.e.*, the formation of e) was also considered, giving a model for a fully condensed IPC-2 zeolite. This structure, as shown in Fig. 5, has only Q^4^ Si species in the interlayer space and so would result in a significant increase in the Q^4^ intensity. Adapting the model to include the formation of structure e leads to a good fit only when *k*_d_ (the rate constant for the step in which e is formed) is significantly lower (∼0.009) than *k*_a_ and *k*_b_ and also lower than *k*_c_, or when unphysical values (*e.g.*, *n*^d^ = 0) are used. This suggests that the production of structure e under these conditions is negligible (∼1%), in agreement with previous work which showed that the production of a fully connected zeolite material requires much higher temperatures (>550 °C)^3,4,10^ than the 50 °C used here (note that as described above such high-temperature treatment corresponds to the “R” step of an ADOR synthesis, rather than the “O” step considered here). Our results indicate that the first two silicon atoms are relatively easy to incorporate into the octets, but intercalation of the third and fourth silicon atoms becomes progressively more difficult to the point where addition of the fourth silicon addition occurs at a negligible rate under the conditions studied here. This is also supported by *ex situ* powder XRD measurements (see Fig. S4.1 of the ESI†) that confirm that the {200} reflection in the reaction product shifts to lower 2*θ* than in IPC-1P, indicating an increase in the average interlayer spacing.^8,10^ However, the *d*_200_ spacing has not yet reached the value characteristic of IPC-2P, suggesting that the reaction to form d has not yet reached completion after 40 h.^8,10^ Although a longer *in situ* reaction would be possible, gelation of the TEOS, owing to the hydrolysis in solution that is seen, would begin to interfere with the experimental measurements (and the formation of Q^2^ oligomers at long reaction times is already starting to be observed). Little or no addition of silicon species to the external surfaces of the crystals occurs during the intercalation process, as confirmed by comparing scanning electron microscopy (SEM) images recorded for the sample before the reaction and after recovery at the end of the *in situ* NMR experiment. These images (see Fig. S4.2 of the ESI†) show no change in morphology of the crystallites and no evidence for the growth of crystalline or amorphous silica on the external surfaces. From the calculated relative amounts of a, b, c and d after 40 h, the extent of reaction is estimated to be ∼50% (as measured from the fraction of possible intercalated silicon atoms), assuming that structure e is not formed at all under these conditions.

**Fig. 7:**
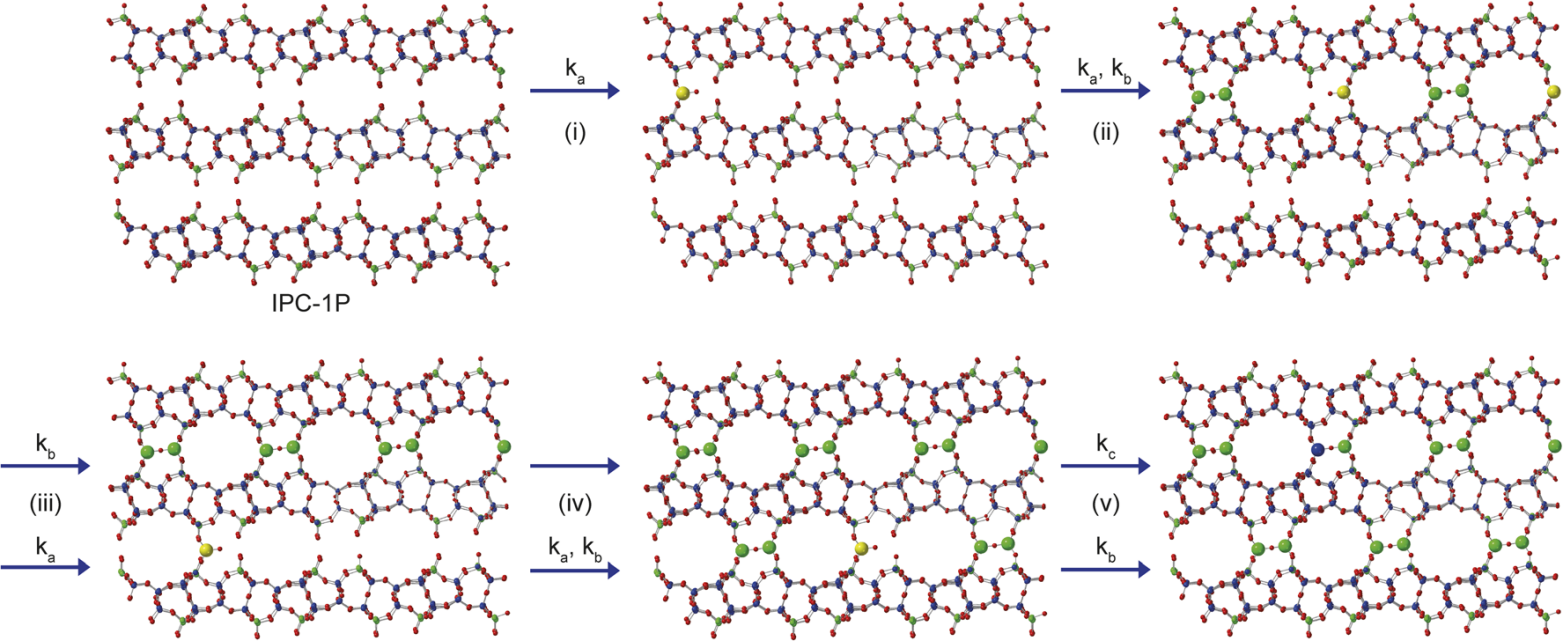
Schematic showing the early stages of the mechanism for the formation of IPC-2P by sequential intercalation of Si(OH)_4_ into IPC-1P. Steps (i) and (ii) show the loss of a to initially form b, and subsequently c, and steps (iii) and (iv) involve further reaction with Si(OH)_4_ within additional layers to form a solid that contains a mixture of b and c. Step (v) contains the much slower reaction to form d in one layer, along with the simultaneous faster formation of c elsewhere. Structures a, b, c and d are defined in Fig. 5. Oxygen atoms are shown in red, and Q^2^, Q^3^ and Q^4^ Si atoms in yellow, green and blue, respectively.

From the results presented above, it is possible to suggest a mechanism to describe the early stages of the reaction of TEOS with IPC-1P, leading to the building of connections between the zeolitic layers (and ultimately to the synthesis of novel zeolitic materials). As shown schematically in Fig. 7, extragallery hydrolysis of TEOS in solution leads to the formation of Si(OH)_4_, which can then intercalate into the IPC-1P layers. Intercalation of the first Si(OH)_4_ molecule (step (i)) needs to be accompanied by an expansion of the spacing between two layers, opening up a zeolite-like channel, which then makes further intercalation of Si(OH)_4_ elsewhere in the layer easier. The similarity of *k*_a_ and *k*_b_ leads to the formation initially of b but also (and as suggested by Fig. 6c), of c, within the layer. Intercalation of further Si(OH)_4_ molecules subsequently occurs within other layers, forming b, and then c, throughout the material. The final step in the reaction is further intercalation of Si(OH)_4_ to form d. The rate constant governing this process, *k*_c_, is lower than both *k*_a_ and *k*_b_, and the process is likely to happen initially at random in layers where structure c predominates. As the reaction continues, the average spacing between the layers will converge towards the characteristic value for IPC-2P.

## Conclusions

The *in situ* monitoring using NMR spectroscopy of one step of the ADOR process has provided both qualitative and quantitative insights into the mechanism and rate of the reactions taking place and helped to improve the understanding of this interesting approach for non-traditional zeolite synthesis. The ability to observe signals from both liquid and solid components is vital to understanding the nature of the intercalating species and the way in which this can react with the layered zeolite starting material. Surprisingly, the hydrolysis of TEOS was shown to occur extragallery (rather than after reaction with the acid catalyst), with Si(OH)_4_ as the intercalating species, in contrast to the expectation from previous work in different systems. Although it is not possible to unambiguously rule out the intercalation of small amounts of any other silicate species into the zeolite, the combination of information from the ^29^Si NMR spectra (*i.e.*, the extremely low levels of Si(OH)_4_ seen relative to previous work on the hydrolysis of TEOS in solution) and from ^1^H NMR spectra (which suggests that ethanol is produced from TEOS hydrolysis only in solution) provides strong evidence for Si(OH)_4_ being the major intercalating species. Future work to investigate this further would be possible (*e.g.*, varying the water content of the reaction), but this would undoubtedly affect the ADOR reaction itself and may well ultimately be limited by the low resolution of the ^1^H NMR spectra, which is limited both by MAS rate for solid-state signals and by the nature of the “slurry-like” sample.

Our NMR experiments are also able to provide unique insight into the molecular level reaction that takes place between Si(OH)_4_ and the octet of silanols in the IPC-1P zeolite precursor, suggesting that the reaction of the first two Si(OH)_4_ molecules occurs at a similar rate (despite the expected energy penalty of separating the layers), and showing that the second Si(OH)_4_ molecule is more likely to bind adjacent to the first, maximising the number of covalent bonds formed, despite the increase in steric hindrance, and rapidly forming structural motif c within each layer. It is clear, however, that the third Si(OH)_4_ molecule to bind to an individual octet reacts more slowly, and there is no evidence (under these conditions) that full condensation occurs to form IPC-2. The final product (after 40 h of reaction) is close to an IPC-2P intermediate. Although a longer reaction could, in principle, result in a higher level of intercalation, the extragallery hydrolysis observed will, ultimately, prevent the reaction reaching completion owing to the formation of oligomeric silicate species which become too large to intercalate into IPC-1P.

This work demonstrates the important role that *in situ* NMR measurements can serve in understanding the local structural changes that take place in the ADOR process with atomic-level detail, and this approach will be vital in the future to gain insight into the different steps in this process and how the mechanism and rate of reaction depend on the experimental conditions (rather than simply understanding the final product formed). These approaches are clearly also applicable to the study of intercalation processes in other heterogeneous solid–liquid systems (*e.g.*, drug adsorption/delivery systems), and the formation and growth of many porous solids, particularly when combined with complementary diffraction studies. There are limitations to the types of reactions that can be studied in this way, with this approach requiring that the reaction happens on a feasible timescale and can be carried out on the scale of the rotor volume and at a temperature that is accessible by the instrumentation. It is also necessary that the spectra acquired have sufficient sensitivity and resolution to observe and assign the species present. In this work, we have exploited isotopic enrichment to enable ^29^Si NMR spectra to be acquired with better sensitivity while retaining good time resolution in the *in situ* study. The cost of this (∼£50 per reaction) does not present any significant barrier to the application of this technique in this instance particularly when compared to the formal cost of instrument time, but the costs and ease of enrichment will vary with the type of system and reaction that is studied. The use of enriched starting materials or reagents also offers additional benefits in terms of the ability to selectively probe specific components of the system, or to follow how and where the intercalating species is incorporated, leading to further mechanistic insights. The isotopic enrichment of the materials that result from the reactions is also of benefit for future spectroscopic studies, potentially enabling the application of more advanced experiments that may not be possible at natural abundance levels. The extraction of quantitative information on the kinetics of the process may require some assumptions to be made about the mechanism that is operating, and for complex reactions or more complex systems will typically involve some simplification, but more qualitative insight should be obtained in all cases. One further limitation may be the need to ensure that both liquid- and solid-state signals can be observed and differentiated. Although we have used single pulse experiments in this work as we required quantitative spectra, the use of cross polarisation to selectively observe the species in the solid is a possible option for systems where more qualitative insight is sufficient. Finally, we also note that the application of MAS may, in principle, influence the system under investigation due to the effects of pressure induced by MAS.^40^ However, for the relatively low MAS frequency used here (5 kHz), the pressure effects are relatively small (they are orders of magnitude lower than the pressures typically required to induce structural changes in “high pressure” solid-state chemistry), but may nevertheless affect the rate or mechanism of some reactions, as indeed occurs due to stirring or tumbling during syntheses. Clearly, the application of MAS also offers a beneficial opportunity to gain insights into the possible pressure dependence of the reaction of interest, by carrying out the *in situ* NMR study at both low and high MAS frequencies.

In conclusion, the simultaneous acquisition of liquid- and solid-state *in situ* NMR spectra (particularly when combined with isotopic enrichment) highlights the great potential to probe, quantify, understand and eventually control non-traditional zeolite synthesis using the ADOR process. Furthermore, the generality of the approach offers future insight in different areas of chemistry and potential for impacting our fundamental understanding of how materials are formed and how they may be utilized in subsequent applications.

## Author contributions

SEA and REM co-conceived the project, had oversight of the supervision of the syntheses, NMR experiments and analysis and co-wrote the final manuscript. NLK and EALB carried out the experimental NMR measurements, performed the kinetic analyses and contributed to the initial draft of the manuscript. GBL and PSW synthesised the enriched zeolite materials used in the work. CEH and KDMH conceived the types of NMR experiments employed, facilitated the *in situ* NMR measurements and analysis, and contributed to the writing of the manuscript. KDMH also contributed to the overall discussions of the results from the project as a whole.

## Conflicts of interest

There are no conflicts of interest to declare.

## Supplementary Material

SC-016-D4SC07931K-s001

## Data Availability

Additional data supporting this article (further details of experimental parameters, spectral analysis, additional results, preliminary DFT calculations XRD/SEM measurements) are provided in the ESI.† The research data supporting this publication can be accessed at https://doi.org/10.17630/4d38d0ea-6238-4ae8-9055-ed92a03979c6.^51^
