# Supplementary material for: Exploiting in situ NMR spectroscopy to understand non-traditional methods for zeolite synthesis
Source: Chem Sci. 2025 Feb 6;16(10):4245–55. doi: 10.1039/d4sc07931k (PMC11803523; doi:10.1039/d4sc07931k)
Supplement: SC-016-D4SC07931K-s001 [file SC-016-D4SC07931K-s001.pdf]

Supplementary Information (SI) for Chemical Science.
This journal is © The Royal Society of Chemistry 2025

Exploiting *In Situ* NMR Spectroscopy to Understand Non-Traditional Methods for Zeolite Synthesis

Nicole L. Kelly,[1] Emma A. L. Borthwick,[1] Gaynor B. Lawrence,[1] Paul S. Wheatley,[1] Colan E. Hughes,[2] Kenneth D. M. Harris,[2]* Russell E. Morris[1]* and Sharon E. Ashbrook[1]*

[1] *School of Chemistry, EaStCHEM and Centre of Magnetic Resonance, University of St Andrews, North Haugh, St Andrews KY16 9ST, UK*

[2] *School of Chemistry, Cardiff University, Park Place, Cardiff CF10 3AT UK*

Supporting Information

S1. Further NMR experimental details

S2. Extraction of integrated intensities

S3. Structure of IPC-1P

S4. Other characterisation of the reaction products

S5. Additional *in situ* NMR spectroscopy

S6. Preliminary DFT calculations

S7. *In situ* ^{1}H NMR spectroscopy

S8. References

S1. Further experimental details

As described in the main text, for the *in situ* NMR experiments, interleaved acquisition of ^{29}Si MAS NMR spectra with recycle intervals of 1 s (averaging 128 transients) and 30 s (averaging 16 transients) was performed over 40 hours, using 90° (3.2 μs) pulses with a radiofrequency nutation rate of ~78 kHz.

In the ideal case (for quantitative measurements) recycle intervals of 5 T_1 are required. However, when studying chemical reactivity, it can often be the case that a compromise has to be reached between complete relaxation and the time resolution required for detailed kinetic analysis. T_1 relaxation times for ^{29}Si species in the initial IPC-1P starting material and the intended final IPC-2P product (although we note this was not completely formed at the end of the experiment), are given (separately for Q^4 and Q^3 species) in Table S1.1. These samples were synthesised from the *ex situ* hydrolysis of Ge-UTL, filtered, washed and dried prior to saturation recovery experiments at 14.1 T (at room temperature). These results show that recycle intervals of 45-60 s would likely be required for the acquisition of quantitative spectra, but it should be noted that (i) these samples have been dried, (ii) the measurements are at room temperature and (iii) the relative relaxation of Q^4 and Q^3 species is very similar in both cases, enabling accurate relative ratios to be obtained even at shorter recycle intervals (note no Q^2 species are present in these materials).

Although we would expect relaxation rates to increase (and T_1 to decrease) during the reaction when the liquid is present and a higher temperature is used, it is very challenging to measure T_1 accurately for the intermediate species present as the system and species are evolving throughout. To estimate these relaxation times, ^{29}Si MAS NMR spectra were acquired for a mixture of IPC-1P and TEOS (at room temperature) at 9.4 T using variable recycle intervals and averaging over 64 transients. Little significant difference was seen in the relative signal intensities for Q^4 and Q^3 Si when recycle intervals above 15 s were used. Although changes in the material are taking place during the measurements, all experiments

were complete within 2 hours (and 80% within 1 hour) with the aim that these changes would be minimised.

When longer recycle intervals were used for other ^{29}Si NMR experiments no additional signals (either in the solid or in solution) were seen, suggesting that the spectra shown here contain signals for all species present at significant levels. Therefore, in order to balance the need for quantitative measurements with good time resolution within the reaction (and noting the similar relaxation times of the Q^4 and Q^3 species), a recycle interval of 30 s was deemed a reasonable choice for the *in situ* solid-state NMR measurements.

Table S1.1. ^{29}Si T_1 relaxation times for the zeolitic materials and systems of interest in this work.

Material	^{29}Si T_1 /s	
	Q^4	Q^3
IPC-1P	12.4 ± 3.4	12.8 ± 3.3
IPC-2P	7.0 ± 2.6	9.5 ± 1.5
IPC-1P+TEOS	<5 [a]	<5 [a]

[a] Estimated from spectra recorded using variable recycle intervals

S2. Extraction of integrated intensities

After acquisition, the interleaved ^{29}Si MAS NMR spectra were reordered to produce separate pseudo two-dimensional datasets for each of the two recycle intervals used. For the data acquired with a recycle interval of 30 s, spectra in the two-dimensional dataset were Fourier transformed and phased. It should be noted that the lineshapes observed for ^{29}Si signals from the solid are not single Gaussians (but instead result from the overlap of multiple types of distinct Si species) and simple analytical fitting is challenging. Therefore, integrated intensities for the Q^4, Q^3 and Q^2 species were determined using numerical integration in MatLab 2019b using the trapz function. Note that for the Q^2 signals, the (low) intensity of the overlapped liquid-state signals from the oligomeric species in solution was subtracted from the total value.

To provide some estimate of the uncertainty in the intensity measurements, integration was also performed manually in Topspin for eight spectra and the absolute difference between the two values was then expressed as a percentage of the MatLab value, and the average taken over the eight spectra. The maximum difference between the two values was ~10% for the Q^4, Q^3 and Q^2 species and is shown as error bars on the plots presented.

This process was repeated for two sets of data; the first contained each spectrum in the data set separately, and the second contained "binned data" (where three consecutive spectra were combined to improve sensitivity, but with the loss of time resolution). In the main text, analysis of only the second set of data is shown, but similar analyses for the first set of data are shown below.

S3. Structure of IPC-1P

Figure S3.1 shows the structure of one layer of IPC-1P,[S1] viewed normal to the layer. This clearly shows the arrangement of the Q^3 Si species (shown in green in Figure S3.1a) and SiOH oxygens (shown in pink in Figure S3.1b) in isolated quartets. In the IPC-1P material adjacent layers come together to form the octets of silanols described in the main text.

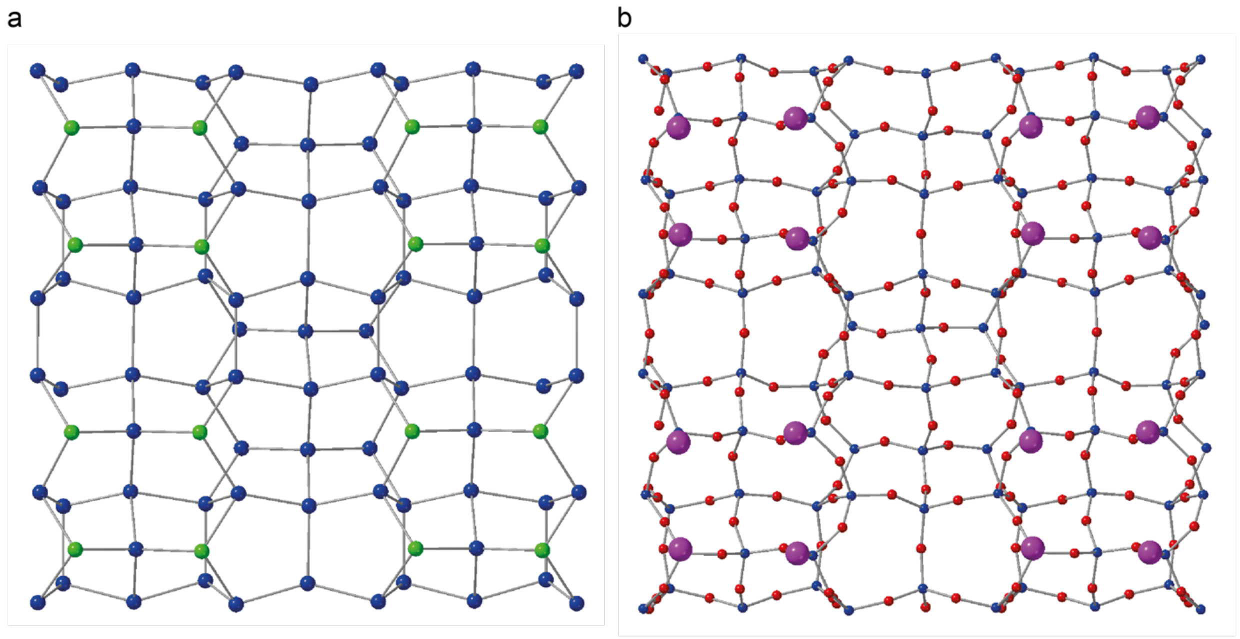

Figure S3.1. Schematic structural models of one layer of IPC-1P (viewed normal to the layer). In (a), the silica framework is shown, with oxygens atoms omitted for clarity (Q^4 Si = blue, Q^3 Si = green). In (b), all atoms are shown (Si = blue, O = red), with oxygens in the SiOH groups indicated as large pink spheres.

S4. Other characterisation of the reaction products

Figure S4.1 shows powder XRD patterns for the parent Ge-UTL zeolite, the IPC-1P starting material, the product of the *in situ* reaction (after extraction from the HRMAS insert and drying) and IPC-2P.[S1-S2] These were obtained using a STOE STADIP instrument operated in capillary Debye-Scherrer mode. For the product obtained after the *in situ* reaction, the d_{200} reflection shifts to lower 2θ than seen in IPC-1P, indicating an increase in the average interlayer spacing. However, comparison to the powder XRD pattern of a sample of IPC-2P synthesised *ex situ* suggests that the interlayer spacing in the *in situ* product has not yet reached the value characteristic of this possible product, and that the reaction has not reached completion under these conditions in 40 hours.

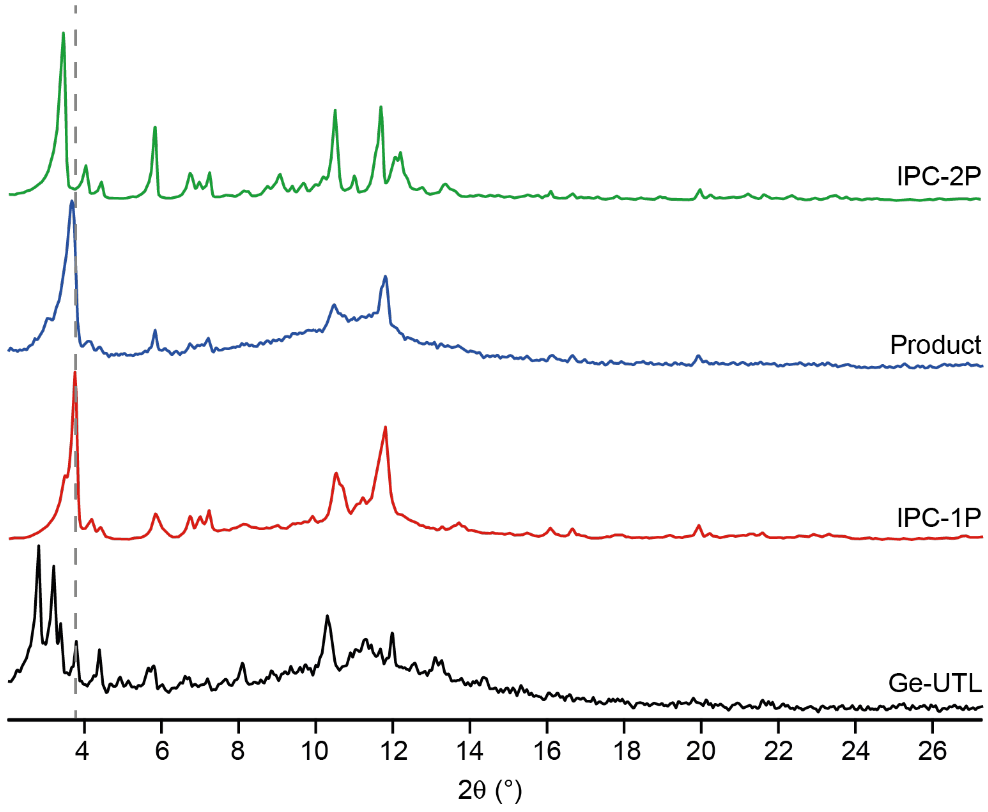

Figure S4.1. Powder XRD patterns (Mo K_{α}) recorded for the parent Ge-UTL zeolite (black), the IPC-1P starting material (red), the product from the *in situ* reaction (blue) and IPC-2P (green). The position of the {200} reflection for IPC-1P is marked with a dotted line.

Figure S4.2 shows scanning electron microscope (SEM) images of the crystallites before (i.e., as IPC-1P) and after the *in situ* NMR experiment. The crystallites show no discernible differences, indicating that there is no significant crystallisation or silica attachment at the external surfaces of the material. The stacked layer/flake motif of IPC-1P is the typical morphology expected for this material, stemming from its synthesis (i.e., selective disassembly of a parent zeolite). These images were obtained using a JEOL JSM-IT200 SEM with a tungsten filament, using a secondary electron detector. Samples were fixed to a SEM stub using C tape and coated in Au using a Quorum Q150R Au/C coater.

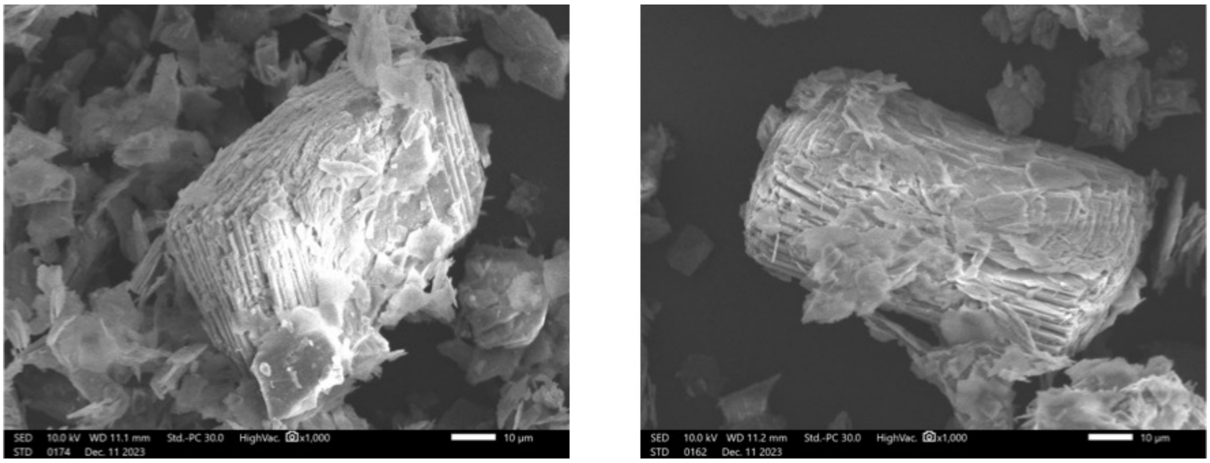

Figure S4.2. SEM images of the crystallites before (left) and after (right) the *in situ* NMR experiment.

S5. Additional *in situ* NMR spectroscopy

Figure S5.1 shows ^{29}Si MAS NMR spectra of the two starting reagents (TEOS and IPC-1P) prior to reaction. There is a significant difference in chemical shift between the TEOS and the signals from the IPC-1P zeolite, as well as a significant difference in linewidth. This enables the two sets of signals from the solid material and the solution to be easily distinguished in the *in situ* experiment.

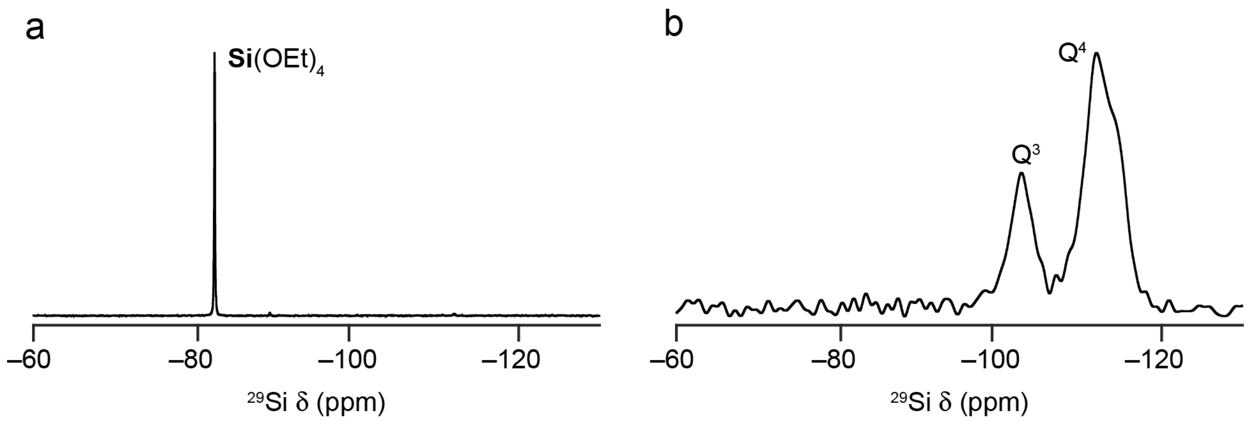

Figure S5.1. ^{29}Si (20.0 T, 5 kHz) MAS NMR spectra of (a) TEOS and (b) IPC-1P.

Figure S5.2 shows an expansion of the sum projection of the two-dimensional dataset in Figure 2a of the main text onto the horizontal axis, enabling the different signals present at different times in the reaction to be seen. This does not represent the real relative intensities of the signals seen at any one point in the reaction. The suggested assignment of the range of oligomeric species seen at longer reaction times is given in Table S5.1. $Q^n(m)$ indicates a Si species with n bonds to Si (i.e., the degree of condensation) and m bonds to OH groups (i.e., the degree of hydrolysis). The symbols △ and □ indicate Si situated in 3 and 4 membered rings, respectively, and X indicates either an OH or OEt group.[S3-S4]

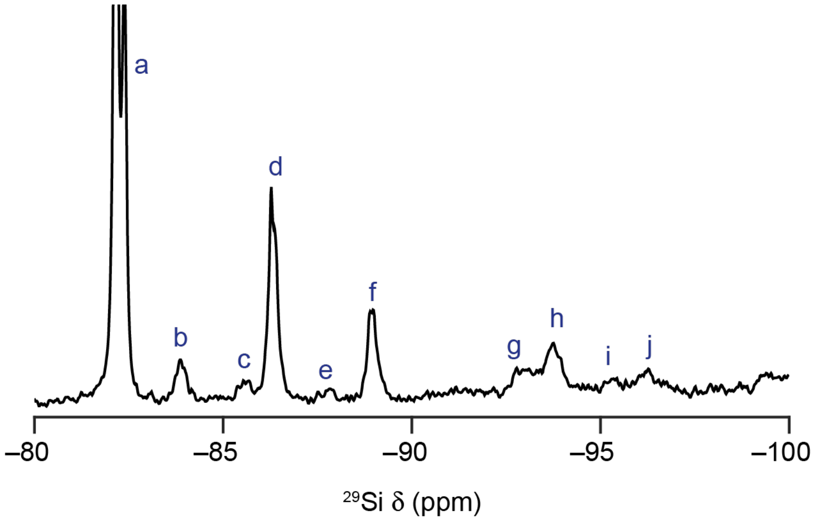

Figure S5.2. Expansion (between –80 and –100 ppm) of the sum projection of the set of ^{29}Si (20.0 T, 5 kHz) MAS NMR spectra acquired during the *in situ* reaction between TEOS and IPC-1P and shown in Figure 2a of the main text.

Table S5.1. Suggested assignment of the signals corresponding to the oligomeric species in the ^{29}Si MAS NMR spectrum shown in Figure S5.2.

δ (ppm)[a]	Label	Suggested assignments	
–82.4	a	$Q^1(3)$	$(OH)_3$**Si**$(OSiX_3)$
–83.4	b	$Q^1(2)$	$(OH)_2(OEt)$**Si**$(OSiX_3)$
–85.5	c	$Q^2{\vartriangle}(1)$	$(OH)(OEt)$**Si**$(OSiX_3)_2$
–86.3	d	$Q^1(1)$	$(OH)(OEt)_2$**Si**$(OSiX_3)$
–87.8	e	$Q^2{\vartriangle}(0)$	$(OEt)_2$**Si**$(OSiX_3)_2$
–88.9	f	$Q^1(0)$	$(OEt)_3$**Si**$(OSiX_3)$
–93.0	g	$Q^2\square(1)$	$(OH)(OEt)$**Si**$(OSiX_3)_2$
–93.7	h	$Q^2(1)$	$(OH)(OEt)$**Si**$(OSiX_3)_2$
–95.3	i	$Q^2\square(0)$	$(OEt)_2$**Si**$(OSiX_3)_2$
–96.2	j	$Q^2(0)$	$(OEt)_2$**Si**$(OSiX_3)_2$

[a] Estimated position of the centre of the signal

Figure S5.3 shows a plot of the % intensity of the liquid- and solid-state signals in the ^{29}Si MAS NMR spectra as a function of time. It can be seen that the % of solid-state signals

increases more rapidly in the first ~5 h of the reaction, with a slower rate of increase after 10 h.

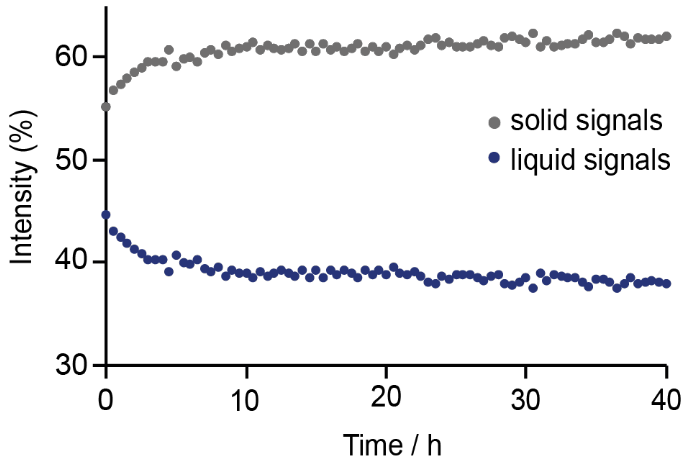

Figure S5.3. Plot showing the intensities (expressed as %) of the liquid- and solid-state signals in the ^{29}Si (20.0 T, 5 kHz) MAS NMR spectra of the *in situ* reaction between TEOS and IPC-1P as a function of time.

Figure 5 in the main text shows the proposed mechanism of intercalation of $Si(OH)_4$ into the layers of IPC-1P and the ratios of NMR-active ^{29}Si Q^4, Q^3 and Q^2 species for each of the structural models **a**, **b**, **c**, **c'**, **d** and **e**. In structure **a**, there are 22 Q^4 species present and 8 Q^3 species (giving the idealised Q^4/Q^3 ratio of 2.75 for IPC-1P). As the IPC-1P zeolite is 18% enriched in ^{29}Si, the number of ^{29}Si Q^4 and Q^3 species is therefore 3.96 : 1.44. The reaction of IPC-1P with an $Si(OH)_4$ species produces structure **b**, converting two Q^3 species into Q^4 species and generating one Q^2 species. As the TEOS is 99% enriched the proportion of ^{29}Si Q^2 species is now 0.99. However, the two additional Q^4 species created are only 18% enriched (and the two Q^3 species lost are only 18% enriched) giving Q^4 : Q^3 : Q^2 of 4.32 : 1.08 : 0.99. The reaction of the second $Si(OH)_4$ generates either structure **c** or **c'**. In **c**, two further Q^3 species in IPC-1P (each 18% enriched in ^{29}Si) are converted to Q^4 species. The Si from the second $Si(OH)_4$ species forms a (99% enriched) Q^3 species as it also binds to the proximate Q^2 Si (which itself is 99% enriched) also converting this species itself to a Q^3 Si. Therefore, the number of ^{29}Si Q^4 species increases to 4.68 (i.e., by 0.18 × 2), while the number of ^{29}Si Q^3 species increases to 2.70 (i.e., with loss of 0.18 × 2 and gain of 0.99 × 2). There are no Q^2 species present in **c**, giving Q^4 : Q^3 : Q^2 of 4.68 : 2.70 : 0. In contrast in **c'**,

the binding of the second $Si(OH)_4$ also results in the conversion of 2 Q^3 species (each 18% enriched in ^{29}Si) to Q^4 species, but now sees the formation of a second Q^2 species (99% enriched). This gives $Q^4 : Q^3 : Q^2$ of 4.68 : 0.72 : 1.98. The binding of the third (99% enriched) $Si(OH)_4$ in **d**, converts a further two Q^3 species in IPC-1P to Q^4 (each again 18% enriched). In the interlayer space there are now two Q^3 and one Q^4 species (no matter whether this reaction is with **c** or **c'**) all of which are 99% enriched, and no Q^2 species remain. This gives $Q^4 : Q^3 : Q^2$ of 6.03 : 2.34 : 0. Finally, binding of the final $Si(OH)_4$ converts a further two (18% enriched) Q^3 species in IPC-1P to Q^4, and results in four Q^4 species (all 99% enriched) in the interlayer space. This gives $Q^4 : Q^3 : Q^2$ of 9.36 : 0 : 0.

The kinetic analysis was carried out using an Avrami-Erofe'ev (JMAK) type approach, varying the k_i and n_i parameters to minimise the difference between the calculated and the experimental (normalised) intensities of the (solid state) Q^4, Q^3 and Q^2 signals in the ^{29}Si MAS NMR spectra. For the binned data, the final values of k_i and n_i extracted from the fitting for the **a** to **b** to **c** to **d** pathway are given in Table 1 in the main text, and the corresponding fits are shown in Figure 6. Similar results for the unbinned data are given in Table S5.2 and Figure S5.4. In both cases, the fitting uncertainties were estimated by varying the parameters to ascertain how this affected the minimised residuals from the fit. For the unbinned data, the scatter and experimental uncertainty are greater, but the values of k_i and n_i obtained are similar and confirm the general conclusions discussed in the main text. As noted in the main text, the Avrami-Erofe'ev equation is likely to be only an approximation, and care should be taken not to overinterpret the parameters and their absolute values.

Table S5.2. The best fit Avrami-Erofe'ev parameters (with estimated uncertainties) for the fits shown in Figure 6 of the main text (binned data) and Figure S5.4 (unbinned data). The units of k_i are h^{-ni}.

Parameter	Binned data	Unbinned data
k_a	0.141(3)	0.141(4)
n_a	0.452(6)	0.463(4)
k_b	0.119(3)	0.121(4)
n_b	0.419(6)	0.428(10)
k_c	0.038(4)	0.010(4)
n_c	0.67(4)	0.97(10)

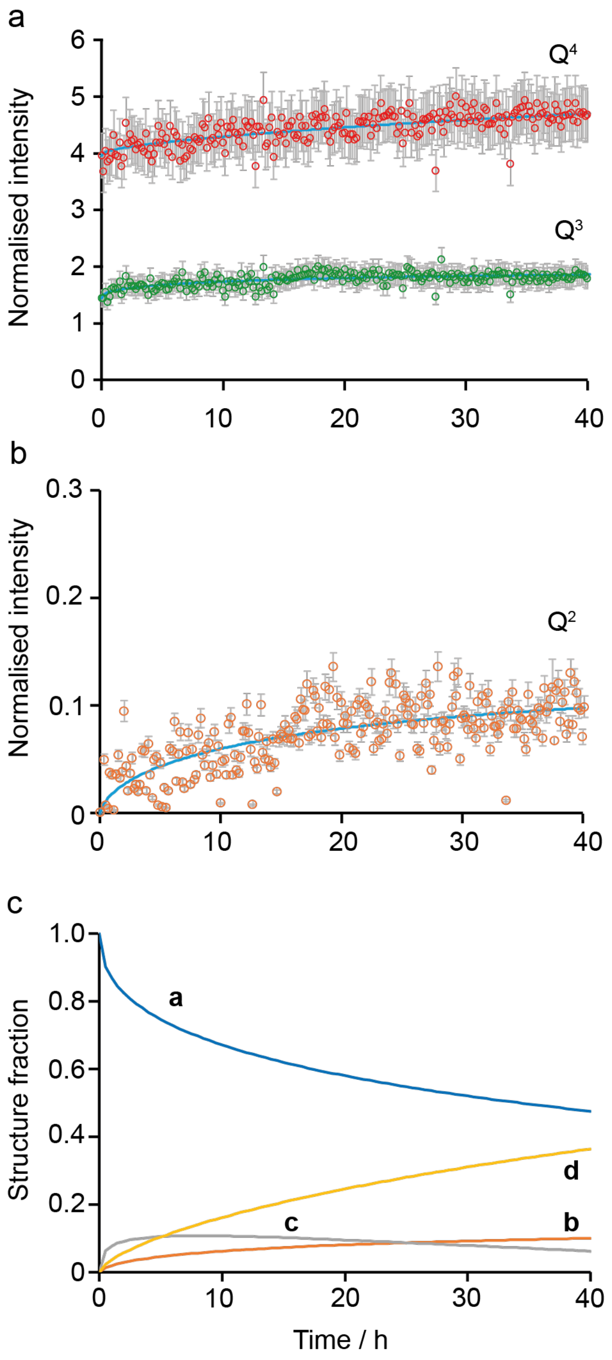

Figure S5.4. Kinetic analysis for the **a** to **b** to **c** to **d** pathway of the reaction mechanism shown in Figure 5. (a, b) Plots showing the fitting of the intensities of the ^{29}Si Q^4, Q^3 and Q^2 signals calculated using the Avrami-Erofe'ev equation (blue lines) to the (normalised) experimental intensities (shown as circles with error bars) extracted from the (unbinned) two-dimensional dataset in Figure 2a. The estimated error bars are ±10% of the experimental value. (c) Plot showing the relative amounts of **a**, **b**, **c** and **d** as a function of time

S6. Preliminary DFT calculations

Preliminary DFT calculations were carried out using the CASTEP DFT code (version 22)[S5] employing the GIPAW[S6] algorithm to reconstruct the all-electron wave function in the presence of a magnetic field. Calculations used the GGA PBE[S7] functional, with core-valence interactions described by ultrasoft pseudopotentials, which were generated on the fly, accounting for scalar relativistic effects by using ZORA. Dispersive interactions were reintroduced using the D3 scheme with Becke-Johnson damping.[S8] A planewave energy cut-off of 60 Ry was used, and integrals over the Brillouin zone were performed using a Monkhorst-Pack[S9] grid with a k-point spacing of 0.04 2π $Å^{-1}$. Models were optimised prior to the calculation of single point energies. A full computational study is beyond the scope of the current work.

Calculations were carried out for three optimised structural models created from (A) the addition of an (unbound) $Si(OH)_4$ unit into the interlayer space of IPC-1P ($Si_{15}O_{32}H_4$), (B) binding of $Si(OH)_4$ to an SiOH group to create a Q^1 species and one molecule of water and (C) binding of $Si(OH)_4$ to two SiOH groups to create a Q^2 species and two molecules of water, as shown in Figure S6.1. While by no means unambiguous models, these initial results do show a thermodynamic driving force for the binding of $Si(OH)_4$ as a Q^2 species rather than a Q^1 species, as shown in Table S6.1, facilitated by the favourable arrangement of the SiOH groups in the octet. In reality, this will also be accompanied by a favourable entropy contribution in each case.

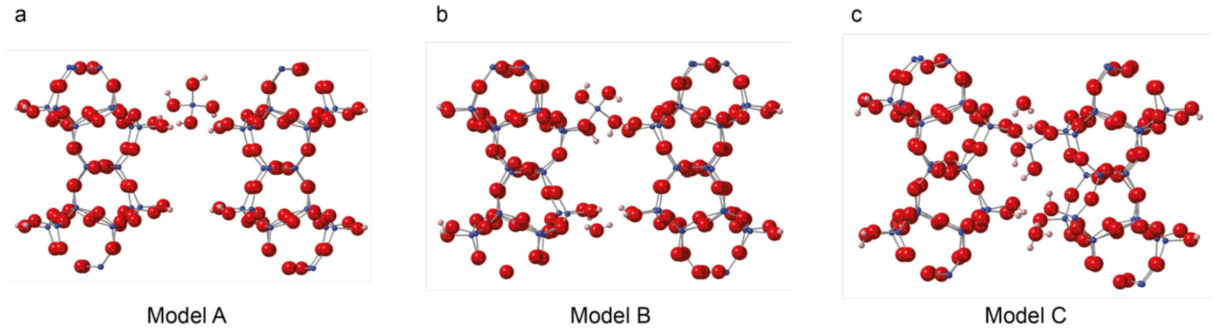

Figure S6.1. Structural models A, B and C used for the DFT calculations described above.

Table S6.1. Calculated enthalpies (ΔH) and enthalpy differences (ΔΔH), quoted per Si, for the structural models A, B and C shown in Figure S6.1.

Model	ΔH per Si / eV	ΔΔH per Si / eV
A	–4599.689	0.000
B	–4599.795	–0.106
C	–4599.894	–0.205

S7. *In situ* ^{1}H NMR spectroscopy

Acquisition of *in situ* ^{1}H NMR spectra was performed both at 14.1 T and 20.0 T by averaging 8 transients with a recycle interval of 3 s every 10 mins over 37.5 h (14.1 T) or 40 h (20.0 T) at 50 °C with an MAS rate of 5 kHz. Spectra were acquired using 90° pulses of 2.5 μs (ν_1 = 100 kHz) at 14.1 T and 3.4 μs (ν_1 = 74 kHz) at 20.0 T.

Figure S7.1 shows ^{1}H (14.1 T, 5 kHz) NMR spectra acquired during the *in situ* reaction of TEOS with IPC-1P as a function of reaction time, with spectra extracted at specific times shown in Figure S7.2b.

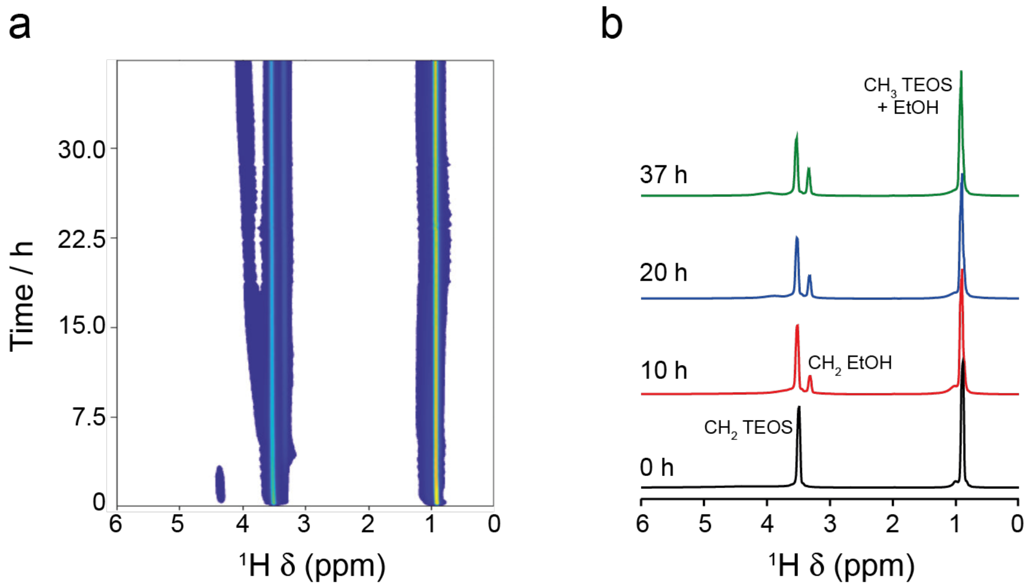

Figure S7.1. ^{1}H MAS NMR spectra (14.1 T, 5 kHz) recorded *in situ* during the reaction of IPC-1P and TEOS loaded into a HR MAS insert inside the NMR rotor. (a) Intensity contour plot of the spectra as a function of time. (b) Spectra extracted from (a) at 0, 10, 20 and 37 h of reaction time.

At the early stages of the reaction, the ^{1}H spectrum is dominated by the signals from the liquid TEOS (at ~0.9 and ~3.6 ppm at 50 °C). There is also signal from the H_2O (at ~4.3 ppm) that was present between the zeolitic layers of IPC-1P but diffuses out to hydrolyse the TEOS. As the reaction progresses, the signal from the zeolitic water is lost (as it reacts with the TEOS), and a new signal from ethanol (CH_2, at ~3.2 ppm) is seen as this is produced

during the TEOS hydrolysis. (Note that the CH_3 signals from TEOS and ethanol are largely overlapped at ~1 ppm). Figure S7.2a shows that the (summed) CH_3 and CH_2 signal intensities remain almost constant throughout the reaction, suggesting that the majority of TEOS hydrolysis (and the formation of ethanol) happens in solution rather than within the zeolitic layers after TEOS intercalation. (Note this is also true for data at 20.0 T, as shown in Figure S7.2b).

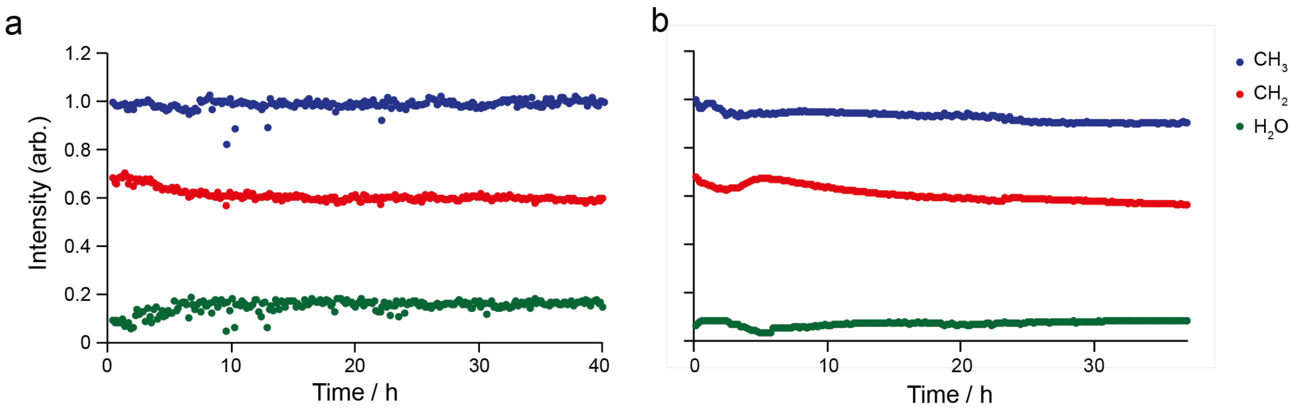

Figure S7.2. Plots showing the variation of the intensities of the three types of ^1H liquid-state signals (CH_3, CH_2 and water) as a function of the time during the *in situ* ^1H NMR study of the reaction of IPC-1P and TEOS at (a) 14.1 T and (b) 20.0 T.

As the reaction between the $Si(OH)_4$ and IPC-1P progress water is formed in the condensation reactions that occur between the zeolitic layers. A new ^1H signal is seen at ~3.5 ppm, which slowly moves to ~4.0 ppm over the 37 h reaction. The ^1H chemical shifts of water and the ethanol OH group will be highly dependent on the nature of the solvent present and the rate of any exchange (with R-OH protons often not observable) and also on the effective pH or acidity. As the reaction progresses the solution composition will vary, with differing amounts of TEOS, water and ethanol at various stages. It seems likely that the new signal that appears is therefore from the water being produced by the condensation (as this appears only later in the reaction and it is clear from the CH_2 signals that ethanol hydrolysis begins much earlier). The ^1H chemical shift of the water then varies as the solvent composition (and hydrogen bonding) and the availability of acid sites on the zeolite (and so the effective pH) vary. The signal from the ethanol OH group is either overlapped with

signals from the water or possibly lost to exchange broadening. Note that the *in situ* ^{1}H spectra are dominated by the much sharper signals from the liquid-state species, as the lines are much broader in the solid state (particularly at low MAS rates). The ^{1}H spectrum of IPC-1P prior to reaction is shown in Figure S7.3 and contains broad and overlapped signals from 2-9 ppm (which will correspond to the SiOH groups that line the layers and the water present within the pores). These signals are not apparent in the *in situ* ^{1}H NMR spectra owing to their much lower peak height intensities.

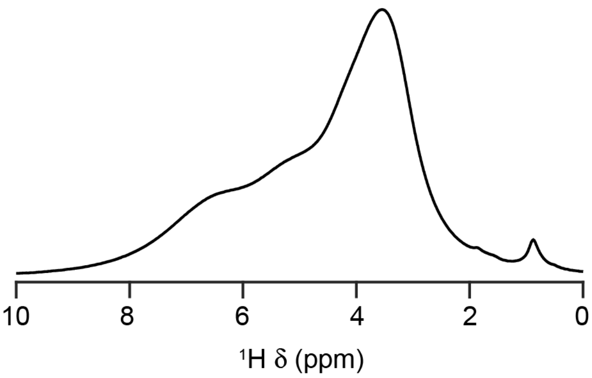

Figure S7.3. ^{1}H (9.4 T, 10 kHz) MAS NMR spectrum IPC-1P.

Although only a preliminary study, it is clear that the ^{1}H spectra are consistent with the hydrolysis of TEOS primarily in solution and the intercalation of $Si(OH)_4$ rather than of TEOS itself. Further future studies would be possible (but ultimately may be limited by both the spectral resolution and the restricted MAS rate).

S8. References

S1. W. J. Roth, T. Sasaki, K. Wolski, Y. Song, D. M. Tang, Y. Ebina, R. Z. Ma, J. Grzybek, K. Kalahurska, B. Gil, M. Mazur, S. Zapotoczny and J. Cejka, *Sci. Adv.*, 2020, **6**, eaay8163.

S2. W. J. Roth, P. Nachtigall, R. E. Morris, P. S. Wheatley, V. R. Seymour, S. E. Ashbrook, P. Chlubná, L. Grajciar, M. Polozij, A. Zukal, O. Shevets and J. Cejka, *Nature Chem.,* 2013, **5**, 628-633.

S3. J. Sanchez, S. E. Rankin and A. V. McCormick, *Ind. Eng. Chem. Res.*, 1996, **35**, 117-129.

S4. C. A. Fyfe and P. P. Aroca, *Chem. Mater.,* 1995, **7**, 1800-1806.

S5. S. J. Clark, M. D. Segall, C. J. Pickard, P. J. Hasnip, M. J. Probert, K. Refson and M. C. Payne, *Z. Kristallogr.*, 2005, **220**, 567.

S6. C. J. Pickard and F. Mauri, *Phys. Rev. B: Condens. Matter Mater. Phys.*, 2001, **63**, 245101.

S7. J. P. Perdew, K. Burke and M. Ernzerhof, *Phys. Rev. Lett*. 1996, **77**, 3865.

S8. S. Grimme, S. Ehrlich and L. Goerogk, *J. Comp. Chem.* 2011, **32**, 1456-1465.

S9. H. J. Monkhorst and J. D. Pack, *Phys. Rev. B* 1976, **13**, 5188.
